# The structural impacts of enforcement policy on Latino immigrant health

**DOI:** 10.3389/fpubh.2022.928435

**Published:** 2022-09-16

**Authors:** Maria-Elena De Trinidad Young, Denise Diaz Payan, Iris Y. Guzman-Ruiz

**Affiliations:** ^1^Department of Public Health, School of Social Sciences, Humanities and Arts, University of California, Merced, Merced, CA, United States; ^2^Department of Health, Society and Behavior, Program in Public Health, University of California, Irvine, Irvine, CA, United States; ^3^Department of Community Health Sciences, Fielding School of Public Health, University of California, Los Angeles, Los Angeles, CA, United States

**Keywords:** immigration enforcement policies, Latino, immigrant health, structural vulnerability, policing, deportation, race/ethnicity

## Abstract

As evidence of the negative health impact of immigration enforcement policy continues to mount, public health research has focused primarily on the psychosocial health mechanisms, such as fear and stress, by which immigration enforcement may harm health. We build on this research using structural vulnerability theory to investigate the structural processes by which enforcement policy may shape Latino immigrants' health. We conducted qualitative analysis of *testimonios* from a purposive sample of Latino immigrants (n=14) living in Southern California in 2015, a period of significant federal, state, and local enforcement policy change. *Testimonios* are a narrative methodology used across the social sciences and humanities to center the voices of marginalized people. Through unstructured *testimonio* interviews, we sought to understand Latino immigrants' experiences with immigration enforcement and identify specific structural factors by which those experiences may influence health. Respondents' narratives revealed that singular enforcement experiences were not viewed as the sole manifestation of enforcement, but as part of a system of intersecting physical, legal, institutional, and economic exclusions which shaped the social and economic conditions that influence health. These exclusions reinforced respondents' marginalization, produced instability about the future, and generated a sense of individual responsibility and blame. We discuss how physical, legal, institutional, and economic processes may influence health and propose a framework to inform population health research on intersecting structural health mechanisms.

## Introduction

A growing body of population health research shows that immigration enforcement policies have a negative impact on the health of individuals, families, and communities (1–4). For example, home and workplace raids and police collaboration with immigrant officials have been found to be associated with substance use (5), poor perinatal health (6–8), and suboptimal child development (9). Existing evidence indicates that the health impact of immigration enforcement can be in part explained by psychosocial health processes, such as chronic stress or healthcare decision-making (2). For example, enforcement policies and the threat of enforcement produces a “chilling” effect, deterring immigrants and their families from accessing federal safety net programs (10–13). Fear of deportations or other enforcement actions can result in immigrant communities experiencing stress, as well as and mistrust of public officials, resulting in delaying or avoiding healthcare services to minimize their risk of detention and deportation (14, 15).

As immigration enforcement has become a sustained presence in immigrants' day-to-day lives, it is critical to expand knowledge beyond the more proximal psychosocial health mechanisms to understand how enforcement policy – a structural force that results in surveillance, policing, and deportation – may shape distal and intermediary pathways to health. In this paper we use a structural vulnerability lens to examine qualitative *testimonios* of Latino immigrants living in California during a period of heightened immigration enforcement. We investigate and identify the structural impacts of immigration enforcement policy that likely influence immigrant health outcomes.

Structural vulnerability theory provides a lens to understand mechanisms between policy and health. Structural vulnerability situates immigrants within “mutually reinforcing insults (ranging from the economic and political to the cultural and psychodynamic)” ((16), p. 4) to assess how enforcement policy may shape the distal, upstream conditions that ultimately influence immigrants' health (17). With this approach, the focus is on individuals' experiences of immigration enforcement *in relation to* social power and inequities as a potential risk to health, rather than a focus on individual-level health processes (e.g., individual health behaviors or decision-making). In contrast to theories on how individual agency or behavior create proximal risks to health (i.e., reactions, coping behaviors), this theory explicitly acknowledges the ‘risk environment’ constraining individuals (18, 19).

Enforcement policies likely contribute to immigrants' vulnerability through multiple structural mechanisms that constrain decision making and frame choices – ultimately shaping more proximal stress and psychosocial mechanisms (20, 21). Prior scholarship applying a structural vulnerability lens has demonstrated how legal status—particularly undocumented status defined and enforced by a country's immigration policy— influences immigrants' well-being through marginalization in social and economic domains and influences how they behave in different environments (21–23). The intersection of structural forces shapes immigrants' marginalized position across multiple levels of social, institutional, and cultural spaces, affecting how individuals cope, how households function, and use social capital (20).

Enforcement policies have long been used to manage the societal membership of racial/ethnic groups, particularly Latinos (24). The 1924 Immigration Act authorized the formation of the U.S. Border Patrol to guard the U.S.-Mexico border to enact patrols, inspection procedures, and deportations, contributing to the production of the prototypical Mexican “illegal alien” (25). The 1996 Illegal Immigration Reform and Immigrant Responsibility Act established the 287(g) provision allowing state and local law enforcement officers to contribute to federal immigration enforcement in local communities (26). By conveying messages, stereotypes, or attitudes regarding “illegality,” policies contribute to ethnic-based inequalities (27), positioning groups in a more structurally precarious position.

Currently, there is a lack of population health research on the potential structural health impacts of enforcement or the social, economic, or other structural mechanisms by which enforcement policies may influence the health and well-being of Latino immigrants. Extensive qualitative research has shown that enforcement actions and policies produce environments that are harmful to mental and physical health (28). The lens of structural vulnerability can build on this knowledge to identify specific structural factors by which enforcement influences health to inform future population health research. In recent decades, the U.S. immigration enforcement system has become extensive and made up of multiple layers of laws and policies, governmental and non-governmental agencies, and on-the-ground practices. Specific policies have changed or evolved to determine the extent of surveillance, policing, and, ultimately, deportation of different immigrant groups (De (29)). Enforcement policy has produced an “intentional and not unusual” (30) system that endorses nativist attitudes and reinforces the subordination of Latino immigrants (31, 32). Understood as a structure that can produce vulnerability, enforcement policy likely produces multiple pathways and processes that influence immigrant health. Future population health research and policy interventions can be informed by an expanded understanding of these structural pathways by which enforcement may influence Latino immigrant health.

In this study, we seek to identify the structural factors that may be mechanisms between enforcement policy and immigrant health through the perspective of Latino immigrants. To apply a structural vulnerability lens, we draw from *testimonios*, a narrative methodology used across the social sciences and humanities to give precedence to the voices of marginalized people (33). Based on these findings, we propose a structural framework that identifies the potential physical, legal, institutional, and economic mechanisms by which immigration enforcement policy may shape health and well-being that can inform ongoing population research.

## Methods

To understand Latino immigrants' experiences with immigration enforcement and identify specific structural factors by which those experiences may have influenced their well-being, we collected and analyzed *testimonios* from Latino immigrants in two counties in Southern California in early 2015. Below we describe the context in which the study was conducted, how we applied a *testimonio* approach to understand Latino immigrants' experiences with enforcement, and how we recruited participants, collected testimonios, and conducted analysis.

### Study context

The policy context during the study's data collection period reflects the multi-level, complex reality of immigration enforcement policy. Nationally, the highest annual number of interior arrests and deportations to date occurred in 2012 (34), due primarily to the Secure Communities program, a federal program to coordinate and share data between local law enforcement and federal immigration enforcement authorities (35). The Deferred Action for Childhood Arrivals (DACA) program was also implemented in 2012 to protect young people who migrated to the U.S. as children from deportation. In 2014, President Barack Obama announced a new deferred action program for parents of U.S. citizens, which provided the possibility of additional protections against deportation for some, but was blocked by the U.S. Supreme Court (36). At the state level, California had recently taken action to limit the impact of federal enforcement through legislation that limited the extent to which law enforcement agencies could transfer individuals who were arrested to immigration officials (37).

### Engaging in the process of *testimonio* to understand Latino immigrants' experiences with immigration enforcement

To understand the experiences of Latino immigrants with immigration enforcement, we engaged in the process of collecting *testimonios* from study participants. *Testimonio* is a process and approach in which a person from a marginalized group orally shares their life experience to bring attention to previously overlooked and often disregarded forms of social inequity (33). The interviewer, who can be a researcher, journalist, or advocate, works to bring the person's words and stories to a wider audience to inform efforts to address those inequities (33). *Testimonio* offered an ideal approach for this study because, first, it recognizes the legitimacy of knowledge held by those who have been marginalized, acknowledging Latino immigrants as a crucial source of knowledge about the health impact of enforcement; and second, it breaks from traditional research approaches which can implicitly or explicitly make assumptions about the issues that matter most to communities, inadvertently otherizing research participants of color (38). The *testimonios* of Latino immigrants provide critical perspectives regarding the negative impacts of enforcement on their own communities.

To apply a *testimonio* approach, we developed an interview guide for unstructured, open-ended interviews. The aim was for the interviews to be respondent-driven conversations, centering Latino immigrants' voices, descriptions, and words that depicted their range of experiences with enforcement policy (39). The interview guide began with a single open-ended question asking the participant to share about themselves. It then included possible topics, such and participants' migration story, their family, their work, or their opinions about immigration policy, that the interviewer could use to invite and encourage conversation about experiences that were most important to the participant.

In developing the interview guide, we chose not to ask participants explicitly about enforcement policy or specific types of enforcement experiences (e.g., if they had ever been detained or deported). We did this to be consistent with a *testimonio* approach in which participants, rather than researchers, shape the narrative. In addition, because of the complexities of enforcement policy, we prioritized participants' own descriptions of experiences, rather than impose our own vocabulary for enforcement policies and actions. As the interviews unfolded, enforcement-related experiences emerged organically as part of participants' overall migration experiences. Similarly, we chose not to ask explicit questions about health to allow participants' own language to describe their understanding and experiences of well-being or illness.

The *testimonio* approach also was appropriate to address ethical concerns about collecting information about sensitive experiences, such as deportation, from a vulnerable study population (40, 41). The study was approved by the Institutional Review Board at the University of California, Los Angeles. Study participants were informed that the study sought to understand how immigration enforcement had influenced their lives and well-being. By approaching the actual interview as an unstructured and open-ended, we sought to develop rapport with participants, ensuring that they only shared sensitive information that they were comfortable with.

### Recruitment

We conducted purposive sampling and recruitment of participants. The aim of this purposive approach was to obtain a sample of individuals with a range of migration and enforcement experiences. To identify potential participants we sought referrals from different sources. We obtained referrals from immigrant rights organizations who worked closely with immigrants, but who were not directly involved in issues related to immigration enforcement and we obtained referrals from one organization whose mission was to provide support to individuals in immigration detention to include individuals who had been directly affected by enforcement. We also obtained referrals from authors' personal networks to identify individuals who were disconnected from service providers.

For each referral, we reached out to contacts at the organization or in our networks and provided them with details about the study. They then inquired among their clients or networks and, after obtaining consent from interested individuals, shared the first name and phone number of interested participants. Author 1 called and screened each participant. While we were unable to make contact with some of the referred potential participants, all who were contacted agreed to participate.

### Collecting testimonios

After being screened by phone, potential participants met with Author 1 in a location of their choosing to learn more about the study, provide verbal informed consent to participate and be recorded, and share their *testimonio* through the unstructured, open-ended interview. Most of the interviews took place in participants' private homes; two took place in a private office at a university; one took place at a church; and one at a coffee shop. The interviews lasted from 1 to 3 hours, were conducted in the language of the participants' choice, in English and/or Spanish, and audio recorded. *Testimonios* used for this analysis included detailed descriptions of individuals' experiences migrating to the United States, establishing new lives, and seeking housing, work, and economic opportunities. Interviewees also described awareness of their subordinate positions and internalized stigma, external enacted stigma (discrimination), decision-making processes, and behavior, which cumulatively, embody the vulnerability (positionality) of Latino immigrants in this study.

After completing each interview, we prepared a memo that documented the most salient topics discussed and identified emerging themes. As described below, after five interviews had been completed, we began an iterative process to develop codes and assess if the interviews had reached saturation. As interviews were completed and iterative analysis proceeded, we determined that we had achieved a saturation of key themes when additional interviews were not yielding new topics or insights.

### Analysis of testimonios

After five interviews had been conducted, we began an iterative analysis process. All interview recordings were transcribed in their original language. First, Author 1 conducted manual line-by-line coding of three of the initial interviews to identify codes that captured unique or discrete experiences. We used *in vivo* codes to allow for respondents' own language to define the categories of analysis. As additional interviews were completed, we iteratively tested codes on additional transcripts to refine the codes and group codes into related topics that captured emerging themes. A final codebook was developed and implemented across all interviews in *Dedoose* (Version 7.5.19) qualitative software. Patterns found across interviews were identified and code trees were created to cluster experiences into four primary themes, described below, that captured both *how* respondents expressed their understanding of enforcement policy and examples of the immediate impact of enforcement on their lives and well-being.

## Results

A total of 14 respondents provided their *testimonio*. Mean age was 40 years (range: 19–59), mean years living in the U.S. was 26 (range: 14–48), and 50% were female (*n* = 7). Respondents were predominantly from Mexico and represented multiple migration trajectories and citizenship statuses (Table 1).

**Table 1:** Characteristics of respondents.

**Name^1^**	**Age**	**Gender**	**Nationality**	**Arrival in US**	**Status**
Aaron	23	M	Mexico	1996	Undocumented and with deportation case pending
Barbara	51	F	Mexico	1989	Undocumented
Eric	53	M	Mexico	1967	Naturalized after gaining legal status under IRCA^2^
Francisco	44	M	Mexico	First in 1989, returned 1996	Undocumented
Hugo	40	M	Honduras	Left US as infant and returned 1997	US Born Citizen
Ines	48	F	Mexico	1989	Undocumented, previously had work permit while trying to obtain U Visa^3^
Jonathan	38	M	Mexico	1979	LPR with deportation case pending
Laura	51	F	Mexico	1989	Undocumented
Lorena	19	F	Mexico	1996	Received DACA^4^
Lucia	24	F	Mexico	2001	Received DACA
Natalie	59	F	Guatemala	1970	LPR^5^ after gaining legal status under IRCA
Oscar	21	M	Mexico	1996	Received DACA
Patricia	40	F	Mexico	First in 1989, returned 1996	Undocumented
Pedro	45	M	Guatemala	First in 1990, deported and returned in 2004	Received withholding of removal^6^ and work permit

1 All names are pseudonyms.

2 Immigration Reform and Control Act of 1986 which, among other provisions, provided legalization for eligible individuals who had resided in the US since 1982.

3 A special visa for individuals who were victims of certain crimes, have suffered mental or physical abuse, and participate in effort to investigate the crime.

4 Deferred Action for Childhood Arrivals, a form of prosecutorial discretion, not a permanent legal status, which defers potential removal for individuals who came to the US as children and meet certain requirements.

5 Lawful Permanent Resident, colloquially referred to as a person who possess a “green card.”

6 An order granted by an immigration judge to an individual who may experience persecution in their home country.

### Enforcement policy, exclusion, and Latino identity

*Testimonios* described the enforcement system as daily, interconnected experiences of physical, legal, institutional, and economic exclusion which, interwoven together, placed respondents in vulnerable circumstances. For respondents, their confrontations with exclusions defined what it meant to be a Latino immigrant in the U.S.– to live in a precarious position and be individually responsible for navigating and overcoming barriers. Singular actions by government agencies (e.g., an arrest) were not viewed as the sole or even primary manifestation of enforcement, but as part of a system of intersecting exclusions. Multiple respondents either directly experienced or were at risk of apprehension or deportation or had family members who were; but even acute consequences of enforcement policy were viewed as part of this larger system. In addition, respondents described the consequences of enforcement policy as barriers they had to navigate with limited social and material support and limited knowledge of the very laws and policies shaping their lives. For example, legal exclusions, such as limited knowledge of immigration laws, unfolded within institutional spaces where racial and ethnic discrimination was common.

### Physical exclusions

Enforcement policy created physical spaces of vulnerability for respondents, particularly for those previously or currently undocumented. The U.S.-Mexico border was respondents' first – and often most memorable – encounter with enforcement policy. Nearly all respondents experienced the challenge and trauma of crossing the U.S.-Mexico border or, for those coming from Central America, the Mexico-Guatemala border. Pedro described one of his journeys migrating from Guatemala to the U.S. via Mexico: “Entering the U.S. was harder that time. Mexican migration was tough, they deported me to Guatemala three times and asked for bribes.” Most respondents entered the U.S. on foot at the U.S.-Mexico border, either crossing in remote areas or official ports of entry where they were concealed or fled from Border Patrol agents and, in some cases, were detained and deported during multiple entry attempts.

Once in the U.S., physical exclusions persisted due to policing by immigration and law enforcement agents. In their day-to-day lives, physical borders manifested themselves in the form of segregated spaces within neighborhoods and geographic regions. Several respondents affirmed that such awareness of their vulnerability in these spaces rarely led them to completely cease daily activities; instead, they navigated physical boundaries by making adjustments to daily patterns. Respondents spoke of concern about local police patrolling or checkpoints. For example, even interactions with police for a potential minor infraction, such as a traffic stop, were viewed as a path to deportation. Growing up undocumented, as Lorena explained, helped her know “where you should and shouldn't drive on a Friday night.” Two participants also talked about avoiding certain stretches on an interstate highway where an interior Border Patrol checkpoint was located.

Deportation represented the most acute physical exclusion, as they were physically removed from the country. The process resulted in feelings of external stigma, suffering, and physical restraint and coercion. Pedro detailed how oppressive and dehumanizing deportation can be, “I was deported to Guatemala on a plane. There were like 280 people. Immigration treats you like you're a murderer. They had us tied at the waist, wrists, and feet the whole way to Guatemala City.” Migration following deportation could also result in physical and emotional trauma for individuals and family members, as described by Eric whose nephew was kidnapped in an attempt to migrate post-deportation: “Two years ago they deported my nephew. I saw how his daughter suffered. But that was not the saddest part. In an attempt to cross back, he was kidnapped for a week and [the kidnappers] called us for a $5,000 extortion.”

### Legal exclusions

Because of their legal status, respondents experienced the enforcement system as an obstacle to having rights, a legal identity, and access to employment and educational opportunities to promote social and economic mobility. Lacking citizenship (as well as legal status) drove uncertainty and insecurity about the future, internalized as a defining aspect of all respondents' Latino immigrant identity. After being detained for 2 years, Jonathan defined citizenship and lack of “American” identity as an understanding of his vulnerability:

Growing up I knew I was never going to be American. But I didn't know that it made a difference to be a citizen…I should have just become a citizen when I turned 18. But I never had the funds. If anything happens [to a citizen], if you get pulled over or arrested you are just going to go to jail and then come back out. You aren't going to get deported or get taken away from what you built here. That's an American.

Proactively seeking and obtaining legal status and, ultimately, citizenship was viewed as critical to assuring security and protections against the enforcement system. The process was a significant source of stress and economic burden for respondents, who were in frequent contact with immigration agencies with hefty fees. Regardless of their current legal status, the experience of being or having been undocumented directly shaped their sense of vulnerability to enforcement, constituting a formative line of exclusion. Respondents had unique trajectories across legal statuses—some had come to the U.S. as children and obtained DACA, one had obtained residency and, eventually, citizenship through the Immigration Reform and Control Act of 1986. All except one respondent had been undocumented at some point in their life, and while some had obtained citizenship, nearly all had experienced a lack of citizenship protections and rights in the U.S., which continued to inform their actions and decision making. All had close family or friends who lacked legal status. For those unable to obtain greater protections, the legal system served as a reminder of the precariousness of their residence in the U.S. Specific efforts and roadblocks are evident in Ines's account:

I used my first paycheck to apply for papers. My daughter has scoliosis, so I hoped to get status because of hardship. Then I applied for a U visa and was rejected. I applied again and was rejected. By then, I had three immigration cases open and the judge kept postponing them. With immigration it's like your hands are tied.

Many respondents had extensive contact and engagement with legal systems, such as the asylum system, immigration courts, and immigration applications or petitions. These legal systems brought respondents into contact with processes – e.g., court hearings, government applications - where they faced binary outcomes, either being granted a legal right to stay in the country or deportation. Often lacking legal support and counsel, respondents described navigating complex laws and bureaucratic processes on their own. Pedro, for example, attempted to apply for asylum and was ultimately deported:

In 1996 I applied for asylum but didn't qualify. The judge said I needed to the leave the country, but I stayed. In 1999 I tried to fix my papers again. Look, in part it was my own ignorance and also the lawyer didn't explain things to me. When I went to court, the judge said, ‘You were supposed to leave in 1996. Now you have an order of deportation.’ They detained me in the courtroom. I was deported to Guatemala on a plane.

For Jonathan, a drug arrest led to him facing the criminal court system with limited knowledge about the ramifications for his legal status. He acquiesced to a plea deal resulting in a felony, making him deportable under immigration law and resulting in self-blame and familial discord:

I took a bad plea deal. The public defender said I'd do 8 months in county, but he didn't say I would have a felony. About a week before I was going to get out of jail a counselor told me [I would be detained]. I called my mom and said, ‘I don't think I'm going to make it.’ If I had known, I wouldn't have taken the plea deal. If my mom had known she wouldn't have let me. She was mad at me. I felt dumb.

### Institutional exclusions

In institutional spaces, respondents contended with discrimination and barriers due to legal status. Overall, while only two respondents had recent, direct contact with ICE, all respondents had experienced high levels of contact with immigration agencies and faced barriers within institutions due to legal status. Because encounters within institutions generally occurred when they were either targeted by an agency official or in the process of seeking services, institutional experiences could be socially isolating and lead to internal stigma and self-blame. Respondents said they felt they had to exercise their rights and seek support to respond to these exclusions, and felt culpable when they failed to do so.

Contact with enforcement agencies ranged from coercive to bureaucratic. Almost all interacted with Border Patrol during the process of migration. Although such encounters transpired many years prior, most still described these as traumatic experiences with long-term effects. Commonplace encounters with agencies linked to enforcement policy occurred frequently through bureaucratic appointments with United States Citizenship and Immigration Services (USCIS) or immigration courts. For instance, Natalie, Ines, and Lorena expressed feeling discriminated against by agency officials at USCIS offices. Lorena, raised in the U.S., said her lighter skin tone and English language skills protected her from more explicit or severe discrimination. In contrast, Natalie and Lorena both felt disrespected because they did not speak English. As Natalie gained fluency in English, she began accompanying and supporting friends to their meetings to provide interpretation to reduce discrimination.

Other institutions included law enforcement, school settings, or social service agencies. While these institutions have distinct organizational goals than those formally tasked with implementing enforcement policies, there were perceived as replicating enforcement-like exclusions by actively imposing eligibility or service restrictions based on legal status or failing to provide protection and support. Institutional policies and practices resulted in direct and indirect exclusions for immigrants in terms of material or social support, benefits, or services. Others expressed mistrust of these institutions and their personnel, thus limiting their willingness to pursue or obtain services.

Most respondents described externally stigmatizing interactions with law and immigration enforcement agencies or internalized a perception that these actors were threats. For some, local law enforcement officers were perceived as enforcers of a punitive system, in contrast to public officials who were viewed as trusted protectors. Lorena reported that where she grew up, “You were taught to be scared of the police.” Oscar shared an experience of being stopped by police officers:

My friend was driving and we were stopped and they asked for my ID, but I didn't want to give it. The officers got mad and one put his hand inside the car to open the door. They started pulling me, but my seat belt was still on. They claimed I had a warrant for arrest and that I had to do a breathalyzer. They arrested my friend.

Occurrences in non-law enforcement settings similarly reinforced a sense of precarity and isolation. Lucia's memory of being in the emergency room for a chronic kidney condition illustrates the link between enforcement policy and institutional policies. Not knowing how to complete intake paperwork requesting a Social Security number, she experienced heightened anxiety about seeking medical care: “I have kidney problems and I remember going to the doctor and on the form in two or three places it asked for a social security. I was afraid and didn't know if they would be able see me. I was feeling so sick, but I didn't want to go and ask the receptionist about what I should put on the form.”

### Economic exclusions

The enforcement system was costly and resulted in economic exclusion, as respondents struggled to cover costs related to the legal system, migration, and deportation. All respondents experienced discrimination and impediments in the labor workforce and in pursuit of higher education—contributing to economic insecurity and uncertainty. For some, such hurdles were attributed to the lack of a Social Security number. Patricia lamented the predicament by saying, “How sad that a piece of paper, one little number, makes such a difference.” Due to legal status coupled with barriers to mobility, work and educational opportunities were constrained. Many dually faced an increased risk of being fired or losing financial aid.

Economic barriers, in turn, exacerbated respondents' barriers to mobility and legal protections or counsel. Multiple respondents shared, for example, that traffic fines for driving without a license caused severe financial burden. Respondents, like Ines, Lorena, Jonathan, and Lucia, faced fees for immigration applications, lawyers, detention bond (used to secure release from the custody of Homeland Security), or *coyotes* (a colloquial term for individuals who guide migrants across the border).

Economic exclusions were particularly internalized as individual responsibility and self-blame. Financial costs, as a result of physical, legal, and institutional exclusions, were generally shouldered by respondents, leading to guilt and stress for individuals and alienation within family structures. As Lucia described, these costs strained her relationship with her father and restricted her family's ability to support him in prison: “Before being deported, my father was in prison for 2 years. It was difficult to visit him because we couldn't drive there, no one had a license. It was expensive to call and mom eventually had to tell him to stop calling because she couldn't afford it. He felt like we didn't care.” For Jonathan, the high bond cost resulted in 2 years of immigration detention while his family worked to pay a lawyer who could advocate to lower his bond:

At first I was given a $50,000 bond. Where was I going to get $50,000? Then my mom got a lawyer, but I had to spend 2 years in detention for the lawyer to get my felony dismissed. Every day I would exercise and watch TV. The deputies were aggressive and rude. They didn't care that immigrants were people, that we were being deported. Finally, the judge lowered the bond to $10,000.

## Discussion

In this study we sought to understand Latino immigrants' experiences with immigration enforcement and identify specific structural factors by which those experiences may influence health. *Testimonios* of Latino immigrants described the U.S. immigration enforcement system as multiple, intersecting forms of exclusion. Beyond fear of any single specific enforcement action, a structural vulnerability lens shows that Latino immigrants continuously confront and navigate the exclusions of enforcement policy—from the initial experience of migrating across the physical political border, to economic precarity as undocumented workers, to navigating legal systems after confronting often discriminatory institutions. The findings in the *testimonios* build on previous studies that have shown that immigration enforcement produces fear and stress, which have been recognized as proximal psychosocial risks to health (2). The findings expand on this knowledge to describe structural impacts by which enforcement policy may produce vulnerability to poor health through physical, legal, institutional, and economic mechanisms. Below we first discuss how our findings correspond with existing research on structural and social determinants of health. We then propose a framework of structural health mechanisms and discuss how these findings can inform understanding of the structural processes by which enforcement policy may influence health.

### The link between physical, legal, economic, and institutional exclusions and immigrant health

The physical, legal, economic, and institutional exclusions identified in this study align with structural and social determinants of health, providing insights into how these factors may influence immigrant health outcomes and directions for future research. As our findings showed, these exclusions had an immediate impact on the broader social and economic conditions in which Latino immigrants made their lives. Exclusions had direct influences on social factors for which there is strong evidence of an impact on health. These include influences on Latino immigrants' neighborhood conditions (e.g., threat of law enforcement in the community), employment opportunities and work conditions (e.g., exclusions from jobs), and educational opportunities (e.g., ability to pursue higher education) (17, 42). Emerging research on immigrant populations suggests that enforcement exclusions have far-reaching implications for the social and economic conditions that shape Latino immigrant health and health care access.

Physical exclusions may produce long-term impacts from repeat or cyclical border crossings on immigrants' legal vulnerability or economic precarity. As the US-Mexico border continues to become more militarized and regulated, there is evidence that encounters with enforcement in the border region are associated with increased stress among Latino immigrants (43). While there is mounting evidence that immigrants' legal status is also a social determinant of health (44), the legal exclusions identified in this study point to the potential health consequences of navigating the numerous legal systems associated with the enforcement system. There are likely mental and physical health impacts of from the challenges of seeking a lawyer, going to court, or fighting deportation. For example, a recent study of immigrants released from detention with remote ankle monitors (used frequently to surveil immigrants with a pending deportation case), identified numerous negative consequences for well-being, such as being isolated from social networks and safety net services (45).

Institutional exclusions likely pose a major barrier to needed health resources. Food insecurity and hunger levels are high among undocumented Latino immigrants. Contending with deportation, along with limited English proficiency (46) can serve as barriers to obtaining resources which could contribute to a household's financial and food security, such as employment (46) or utilization of available public assistance (47, 48). Recent research shows that immigrants and their family in anti-immigrant policy contexts are at risk of avoiding public benefits. One study found a decline in enrollment of children in Medicaid in communities with greater enforcement activity (13); after the announcement of the public charge rule, there was a similar decline in enrollment of children in Medicaid (49). Finally, economic exclusions likely compound Latino immigrants' existing economic precarity and disadvantage. For example, a recent study in Arizona found an immigration arrest resulted in an average of $24,000 in associated costs for families (50). Research is needed to assess the how the costs of being affected by the enforcement system – from traffic fines to lawyers fees to paying detention bonds - affect health outcomes.

Future health research can bridge qualitative methods with population and epidemiologic methods to examine and assess how immigrants' navigation of specific exclusions (i.e., physical, legal, institutional, and economic) may be associated with particular health outcomes. This research can inform demographic, population, and health services research. For example, quantitative surveys and administrative data can develop new measures to assess indicators of physical, legal, economic, and institutional exclusion, moving beyond indicators of individual behaviors or responses to understanding population-level structural processes by which enforcement policy may shape health.

### A framework of structural mechanisms between enforcement policy and immigrant health

**Figure 1:**
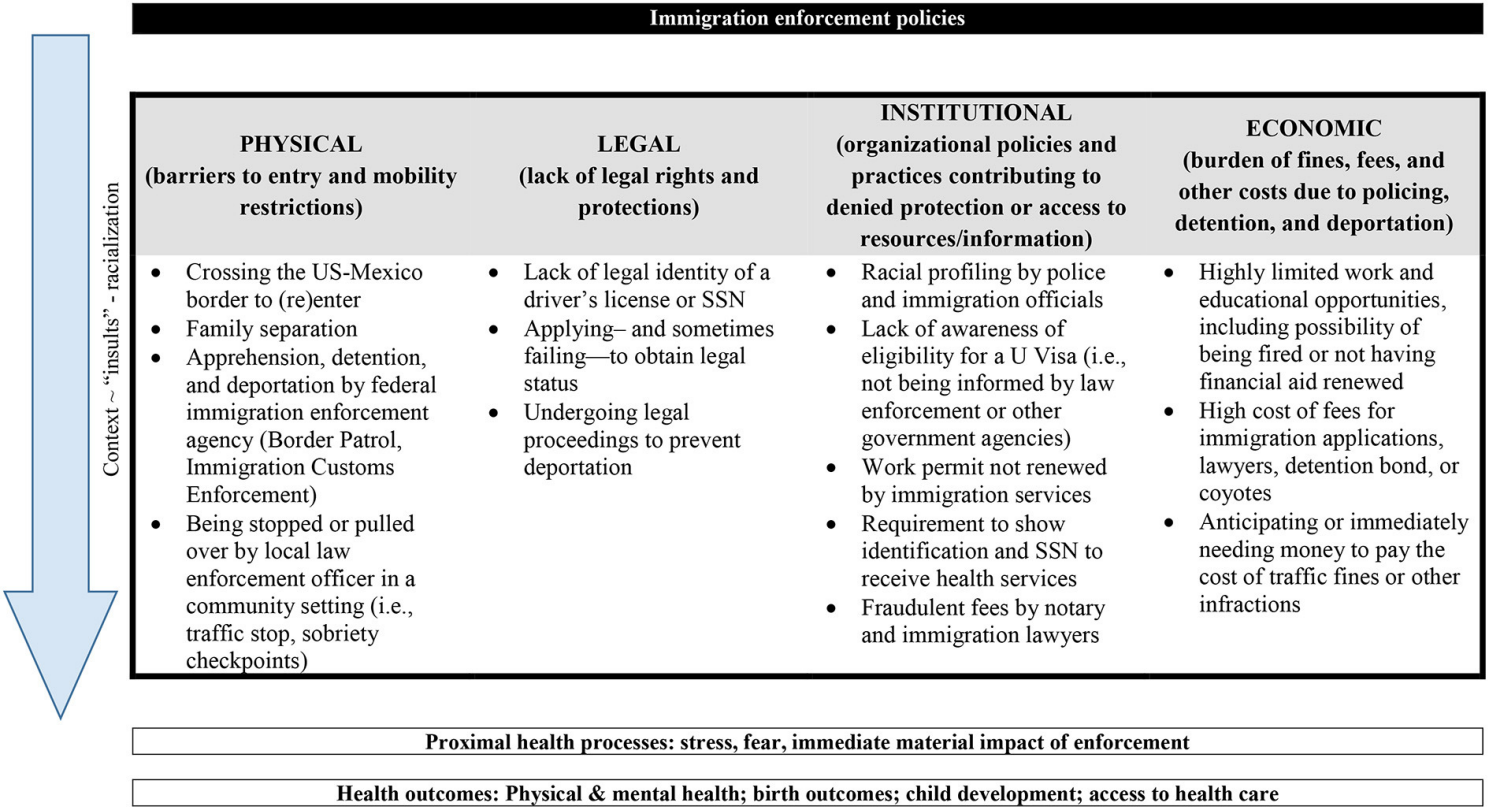
A framework of the structural impacts of enforcement policy on Latino immigrant health.

Toward informing future research, Figure 1 presents a framework that brings together intersecting enforcement exclusions that create structural processes - or mechanisms - to influence immigrant health outcomes. Immigration enforcement results in stress, fear, and direct punitive impacts (e.g., deportation) for immigrant populations, processes shown to influence health (22). Consistent with a social determinants of health approach (17), the framework, goes “upstream” from proximal processes to identify the intersecting physical, legal, institutional, and economic impacts that likely influence proximal health processes and by which immigration enforcement policy may influence health. The framework describes the physical, legal, institutional and economic exclusions identified in the study as co-occurring processes that may influence health at a structural, distal level by determining Latino immigrants' day-to-day contexts, exposing them to the health “insults” of exposure to specific enforcement actions, and, ultimately shaping their sense of identity as Latino immigrants in the US.

First, the framework draws on the *testimonios* to illustrate that enforcement policy creates a context in which immigrants must mitigate risk, adapt, and cope. Consistent with ethnographic health studies that show enforcement and policing practices as creating “pathogenic” environments in which immigrants contend with the consequences of chronic stress and may delay seeking needed health care (51), we found that no single event or encounter exclusively defined respondents' description or experience of enforcement policy. Instead, respondents described lives contending with enforcement policy exclusions as pervasive and commonplace experiences. For example, the enforcement system was described as a host of institutions. Enforcement actions, such as checkpoints, detention, or deportation, were not perceived as distinct from the broader system of immigration laws and policies, such as the process for obtaining legal status or limited rights and protections of the undocumented. Further, the enforcement system was not perceived to be limited to enforcement-specific institutions, but included government agencies such as USCIS, lawyers and notaries, and, even in some cases, health and social service agencies. Consistent with research on how public institutions produce “administrative burdens” on individuals, we found that these non-enforcement institutions ultimately compounded the barriers to health resources due to the complexities surrounding eligibility, enrollment, and access to their resources (52).

Second, the framework brings attention to the potential health “insults” due to enforcement policy described in the *testimonios*. A structural vulnerability lens focused on describing the structures and systems that produce these constraints to achieving health, showing that respondents did not describe the potential harms of enforcement policy as an individualized or psychologized experience. Rather, respondents described living and contending with layered exclusions that produced precarity—from their ability to pay bills or remain in the country, to external stigma imposed by government actors, to limited employment opportunities. That precarity then resulted in proximal experiences of stress or barriers to needed resources. Through this process, the structural production of health vulnerability becomes “embodied” (16). The framework includes examples pulled from the *testimonios* that can inform future studies seeking to understand population-level experiences of these health “insults” within the structural context of enforcement policy.

Embedded in the framework are also the processes by which navigating physical, legal, institutional, and economic barriers may shape Latino immigrants' understanding of their racialized position living in an era of increased enforcement. In the *testimonios*, these exclusions informed participants' sense of what it meant to live as a Latino immigrant in the U.S. Consistent with other studies that have demonstrated the racializing impact of different immigration and citizenship policies (53), these exclusions reflected Latino immigrants' experiences of interpersonal discrimination and structural racism. The racialized nature of these exclusions may harm health through discrimination and racism. Existing evidence shows that Latino immigrants' barriers to health care and exposures to chronic stress as they navigate daily lives (54, 55). Findings also suggest that, despite enacting agency, exclusions reinforced respondents' sense of isolation and individual culpability. Respondents' experiences were isolating and framed as individually inevitable, rather than the product of structural forces.

As Latino and other immigrants continue to contend with the ever-changing nature of enforcement policy in the United States, this proposed framework provides a starting point for continuing to advance knowledge about how surveillance, policing, and deportation determine immigrants' position and their vulnerability to poor health. This study, however, has certain limitations. First, as a respondent-driven study, there was little explicit discussion of health status or outcomes. Therefore, the proposed mechanisms require future study to test their relationships with specific health outcomes. Second, the *testimonios* analyzed here were selected purposively for the specific context in which respondents were living but are not representative of the nation's diverse Latino immigrant population. Future studies can identify other structural mechanisms in other regions and different time periods. For example, concerns around enforcement specific to workplace conditions did not emerge as a significant theme and this may differ in a sample with Latino immigrants living in rural, agricultural regions.

## Conclusion

Through voices of Latino immigrants, this study identifies multiple mechanisms by which enforcement policy may influence health. Knowledge on enforcement policy and health continues to be critical to inform policy change and community-based responses to protect the well-being of Latino immigrants. A structural lens can advance this base of knowledge through identification, measurement, and assessment of the unique and intersecting influence of physical, legal, economic, and institutional exclusions by which enforcement policy shapes Latino immigrants' lives and well-being.

## Data availability statement

The datasets presented in this article are not readily available because Data cannot be shared to protect respondent privacy and confidentiality. Requests to access the datasets should be directed to mariaelena@ucmerced.edu.

## Ethics statement

This study was reviewed and approved by UCLA Office of the Human Research Protection Program. Written informed consent for participation was not required for this study in accordance with the national legislation and the institutional requirements.

## Author contributions

M-EDTY conceived the study, collected and analyzed the data, and contributed to the manuscript. DDP and IG-R contributed to the analysis and manuscript. All authors contributed to the article and approved the submitted version.

## Funding

This paper was funded in part by National Institute on Minority Health and Disparities (R01 MD012292).

## Conflict of interest

The authors declare that the research was conducted in the absence of any commercial or financial relationships that could be construed as a potential conflict of interest.

## Publisher's note

All claims expressed in this article are solely those of the authors and do not necessarily represent those of their affiliated organizations, or those of the publisher, the editors and the reviewers. Any product that may be evaluated in this article, or claim that may be made by its manufacturer, is not guaranteed or endorsed by the publisher.

## Author disclaimer

The content is solely the responsibility of the authors and does not represent the official views of the National Institutes of Health.
